# Inotuzumab ozogamicin for the treatment of adult acute lymphoblastic leukemia: past progress, current research and future directions

**DOI:** 10.1186/s13045-024-01552-7

**Published:** 2024-05-11

**Authors:** Nicholas J. Short, Elias Jabbour, Nitin Jain, Hagop Kantarjian

**Affiliations:** Department of Leukemia, The University of Texas MD Anderson Cancer Center, Unit 428, 1515 Holcombe Boulevard, Houston, TX 77030 USA

**Keywords:** Antibody-drug conjugate, Clinical trials, Efficacy, Lymphoid diseases, Immunotherapy

## Abstract

Inotuzumab ozogamicin (INO) is an anti-CD22 antibody-drug conjugate that was first evaluated in B-cell lymphomas but was subsequently shown to be highly effective in acute lymphoblastic leukemia (ALL). INO improved response rates and survival in a randomized study in adults with relapsed/refractory B-cell ALL, leading to its regulatory approval in the United States in 2017. While the formal approval for INO is as monotherapy in relapsed/refractory ALL, subsequent studies with INO administered in combination with chemotherapy and/or blinatumomab both in the frontline and salvage settings have yielded promising results. In this review, we discuss the clinical development of INO in ALL, highlighting lessons learned from the initial clinical trials of INO, as well as the many ongoing studies that are seeking to expand the role of INO in ALL.

## Introduction

**Fig. 1:**
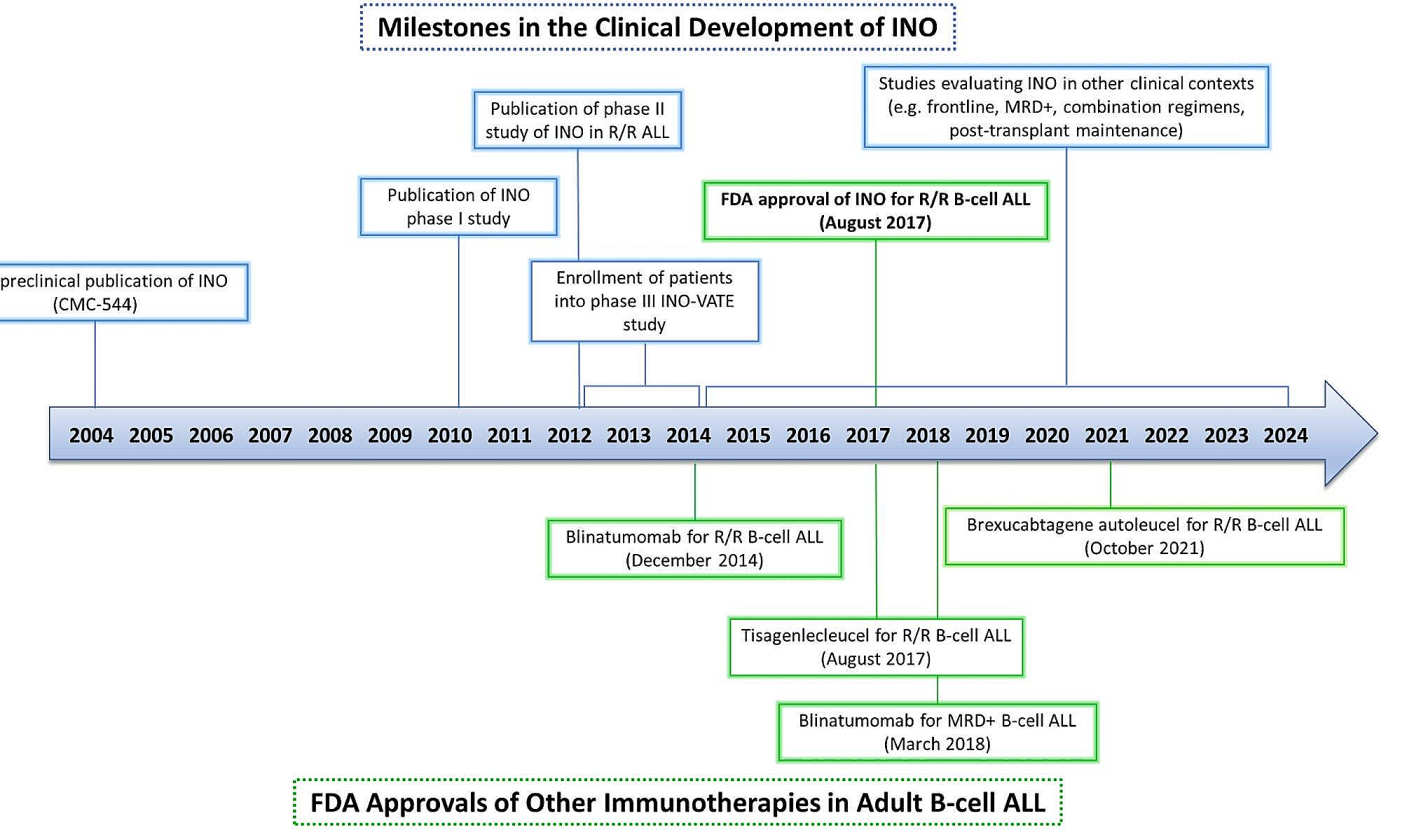
Timeline of the clinical development of inotuzumab ozogamicin in acute lymphoblastic leukemia. For context, approval dates for other novel immunotherapies in adult B-cell acute lymphoblastic leukemia are also shown

The anti-CD22 antibody drug conjugate inotuzumab ozogamicin (INO) was developed in the early 2000s based on initial preclinical data showing promising activity in B-cell lymphoid diseases. These laboratory observations were then followed by several early phase clinical trials that showed significant efficacy of INO in acute lymphoblastic leukemia (ALL), ultimately prompting to its evaluation in a large, randomized trial in adults with relapsed/refractory CD22-positive B-cell ALL. In the pivotal INO-VATE study, INO significantly improved response rates and overall survival (OS) compared with conventional chemotherapy, leading to its approval by the Food and Drug Administration (FDA) in August 2017 [1]. Figure 1 shows a timeline of its clinical development. In this review, we discuss the lessons learned during its development and how these are being applied to current research efforts. We will also discuss the new research that is attempting to expand the potential applications of INO in B-cell ALL, including using it in combination with chemotherapy and/or other immunotherapies, in the frontline treatment of ALL, and in treatment of measurable residual disease (MRD).

### Drug mechanism and preclinical development

INO is an IgG anti-CD22 monoclonal antibody drug conjugate that was developed by Celltech (a British biotechnology company) and Wyeth (a pharmaceutical company, later purchased by Pfizer in 2009). It is covalently linked to calicheamicin dimethyl hydrazide with acid-labile 4-(4’-acetylphenoxy) butanoic acid liner [2]. INO has sub-nanomolar binding affinity to CD22 and is rapidly internalized upon binding, after which it delivers the calicheamicin toxin intracellularly where it binds to the minor DNA groove and leads to double-strand cleavage and subsequent apoptosis. INO was first shown in preclinical studies to be active against B-cell lymphoma cell lines [2]. Subsequent studies were performed in mouse models of aggressive B-cell lymphomas, showing both monotherapy activity as well as synergy with rituximab or chemotherapy, including CVD and CHOP [3–5]. Given the clear preclinical activity in B-cell lymphoma models, INO was also tested in CD22-positive ALL models, where it induced complete tumor regression and cures in mice, warranting its clinical development in ALL [5, 6]. 

### Phase I and II studies

The first study of INO in humans was a phase I study in adults with relapsed or refractory CD22-positive B-cell non-Hodgkin’s lymphoma [7]. Seventy-nine patients were treated, and the maximum tolerated dose (MTD) was 1.8 mg/m^2^ administered as a single dose every 3–4 weeks. Thrombocytopenia was the dose-limiting toxicity, with 90% of patients experiencing thrombocytopenia of any grade, which was grade ≥ 3 in 63%. Encouraging activity was observed, and the overall response rate was 39% among all patients, with response rates in follicular lymphoma and diffuse large B-cell lymphoma of 69% and 15%, respectively, at the MTD. Investigator-initiated pilot studies at MD Anderson Cancer Center were ongoing simultaneously, though the chosen regulatory approval path by the company was initially in lymphomas. Fortunately, by the time the phase III pivotal trial in lymphoma had failed to meet the primary study endpoint in 2014 [8, 9], the pilot studies in ALL had shown encouraging results, thus shifting the regulatory focus to ALL.

The investigator-initiated phase II study at MD Anderson Cancer Center evaluated INO in children and adults with CD22-positive relapsed or refractory ALL (Table 1). In the initial publication, 49 patients received INO at a dose of 1.3 mg/m^2^ to 1.8 mg/m^2^ administered once every 3–4 weeks [10]. The population was heavily pretreated, with 73% of patients being treated as second or later salvage. The complete remission (CR)/CR with incomplete hematologic recovery (CRi) rate was 57%, and the median OS was 5.1 months. The most common adverse events were fever (59%), transaminase elevation (57%), and hyperbilirubinemia (29%). An important observation was that allogeneic hematopoietic stem cell transplantation (HSCT) increased the risk of toxicity. Among the 26 patients who underwent HSCT following INO, the 1-year OS rate was only 20%, driven by higher rates of non-relapse mortality (NRM) and 5 deaths due to sinusoidal obstruction syndrome (SOS) / veno-occlusive disease (VOD). To improve upon the safety/efficacy profile of INO, the study was then amended to fractionate the dose of INO and administer a dose of 0.8 mg/m^2^ on day 1 and 0.5 mg/m^2^ on day 8 and 15, given every 3–4 weeks, with the rationale that lower dose and more frequent schedules of INO may improve anti-ALL efficacy (which is determined primarily by the area under the curve) while reducing toxicities (which is determined primarily by the peak level of INO). In a subsequent analysis after treating 90 total patients (49 at the original schedule and 41 at the new schedule), the response rates and survival outcomes were similar [11]. However, the new dosing schedule appeared safer and resulted in lower rates of fever, hypotension and hyperbilirubinemia. The rate of SOS/VOD was also lower with the new schedule (7% versus 17% with the previous schedule), which may have been driven by the fractionated dosing as well as better understanding of the SOS/VOD risk with INO, leading to a reduced use of alkylating agents in HSCT preparative regimens.

**Table 1:** Early clinical trials evaluating inotuzumab ozogamicin monotherapy in adults with relapsed/refractory B-cell ALL

Study	*N*	Age, median [range]	INO dosing	CR/CRi Rate	HSCT Rate	Median OS	VOD/SOS rate
Kantarjian H et *al*. 2013 [11] (Phase II)	90	39.5 [4–84]	• *Patients #1–49*: 1.3–1.8 mg/m^2^ once per cycle • *Patients #50–90*: 0.8 mg/m^2^ on D1, 0.5 mg/m^2^ on D8 and D15 each cycle	58%	40%	6.2 months	17%^a^
Deangelo DJ et *al.* 2017 [12] (Phase I/II)	72	45 [20–79]	• *Induction*: 0.8 mg/m^2^ on D1, 0.5 mg/m^2^ on D8 and D15 each cycle^b^ • *Once in CR/CRi*: 0.8 mg/m^2^ on D1, 0.4 mg/m^2^ on D8 and D15 each cycle	68%	33%	7.4 months	6%
Kantarjian H *et al.* 2016 [1] (Phase III)	164^c^	47 [18–78]	• *Induction*: 0.8 mg/m^2^ on D1, 0.5 mg/m^2^ on D8 and D15 each cycle • *Once in CR/CRi*: 0.5 mg/m^2^ on D1, D8 and D15 each cycle	80.7%	41%	7.7 months	11%

Abbreviations: INO, inotuzumab ozogamicin; CR, complete remission; CRi, complete remission with incomplete hematologic recovery; HSCT, hematopoietic stem cell transplant; OS, overall survival ; VOD/SOS, veno-occlusive disease / sinusoidal obstruction syndrome

^a^ VOD/SOS rate only reported in transplanted patients

^b^ Phase II doses are shown in the table. Phase I of the study evaluated induction INO doses of 1.2–1.8 mg/m^2^ given in divided dosing

^c^ Data refer only to patients randomized to the INO arm

The safety and efficacy of INO was later confirmed with a phase I/II multicenter study that evaluated INO in a similar population of adults with relapsed or refractory ALL (Table 1) [12]. This study also evaluated divided, weekly doses of INO (ranging from 1.2 mg/m^2^ to 1.8 mg/m^2^ per cycle) given for up to 6 cycles. The recommended phase II dose was 1.8 mg/m^2^ per cycle, with the dose reduced to 1.6 mg/m^2^ once CR/CRi was achieved. Seventy-two patients were treated, including 78% in salvage 2 or beyond and approximately one-third who had undergone previous allogeneic HSCT. The CR/CRi rate was 68% (including CR in 32%), and the median OS was 7.4 months. One-third of patients received a subsequent allogeneic HSCT, and there were 4 cases of SOS/VOD (6% total).

### Phase III study (INO-VATE)

#### Efficacy and safety outcomes

Based on the promising safety and efficacy data from the 2 prior clinical studies of INO in B-cell ALL, the INO-VATE study was designed as pivotal trial to compare INO to conventional chemotherapy in adults with relapsed or refractory CD22-positive B-cell ALL (Table 1) [1]. Three hundred and twenty-six patients were randomized 1:1 to INO or combination chemotherapy (either fludarabine, cytarabine and granulocyte-stimulating factor [FLAG], cytarabine plus mitoxantrone, or high-dose cytarabine). Given the superior safety observed with weekly dosing, INO was given at a dose of 0.8 mg/m^2^ on day 1 and 0.5 mg/m^2^ on days 8 and 15, for up to 6 cycles. The median age was 47 years in both arms, and 32% of patients in the INO arm and 36% in the control arm were in second salvage. INO resulted in a significantly higher rate of CR/CRi than did conventional chemotherapy (80.7% [95% confidence interval (CI), 72.1–87.7%] vs. 29.4% [95% CI, 21.0–38.8%], respectively; *P* < 0.001). Superior responses with INO were observed across all subgroups, with the exception of patients with t(4;11), although the number of patients was small. Among responders, INO was also associated with significantly higher rates of MRD negativity by multiparameter flow cytometry (78.4% vs. 28.1%, respectively; *P* < 0.001) and higher rates of subsequent HSCT (41% vs. 11%, respectively; *P* < 0.001). Driven by the higher rates of response and HSCT realization, INO resulted in significantly better median OS (7.7 months [95% CI, 6.0 to 9.2] vs. 6.7 months [95% CI, 4.9 to 8.3]; *P* = 0.04). While the numerical improvement in median OS was marginal, the greatest benefit to INO was observed in the long-term survival outcomes, where INO more than doubled the 2-year OS rate compared with chemotherapy (23% vs. 10%, respectively). Febrile neutropenia and thrombocytopenia were more common in the control group, while liver-related adverse events were more common with INO. The SOS/VOD rate with INO and chemotherapy were 11% and 1%, respectively. Based on the substantial improvement in both response rates and OS, the FDA approved INO in August 2017 for the treatment of adults with relapsed/refractory B-cell ALL.

#### Subgroups analyses, including transplant outcomes

Following the initial publication of the INO-VATE study, several subgroup analyses of the trial population have been published. These analyses have highlighted important considerations for the use of INO, including its good activity irrespective of bone marrow blast percentage, extramedullary involvement, or CD22 expression, and its activity in Philadelphia chromosome (Ph)-positive ALL [13–15]. INO is associated with a higher rate of HSCT realization, which is the most significant predictor of OS following INO therapy by multivariate analysis [16]. Among patients in the INO-VATE study who received INO and achieved CR/CRi, those who underwent subsequent allogeneic had the best outcomes (median OS 12.6 months and 2-year OS rate 39% versus median OS 7.1 months and 2-year OS rate 13% in non-transplanted). However, subsequent transplant is associated with higher risk of SOS/VOD after INO (23% versus 9% in non-transplanted patients), which contributes to INO-related non-relapse mortality. Proper patient selection for INO and mitigation strategies are therefore imperative to prevent this important potential copmlication. Similar post-transplant findings were observed in a pooled analysis of 2 INO studies, where patients who underwent allogeneic HSCT following INO had a post-HSCT median OS of 9.2 months and 2-year post-HSCT OS rate of 41% [17]. The overall rate of SOS/VOD among transplanted patients across these 2 studies was 18%.

Pooled analyses from multiple INO studies have been used to better understand the risk for SOS/VOD, which is a severe and potential toxicity with INO treatment. Across these studies, the predictors for the development of SOS/VOD include: older age, the use of double alkylator preparative regimens for HSCT, elevated pretreatment transaminases and/or bilirubin, more cycles and higher cumulative doses of INO, and multiple prior ALL therapies, especially prior HSCT [1, 18–20]. Subsequent consensus guidelines have been developed to mitigate these risks. Important considerations to prevent the risk of SOS/VOD in patients receiving INO include: proper selection of patients (e.g. avoiding in patients were severe underlying hepatic dysfunction, avoiding dual alkylator conditioning regimens in transplanted patients, limiting INO to a cumulative dose of 2.7 to 3.6 mg/m^2^ in patients proceeding to allogeneic HSCT, use of high dose steroids at the first sign of liver dysfunction, and distancing the last dose of INO from time of HSCT [21]. Ursodiol prophylaxis 300 mg three times daily should be considered for all patients receiving INO, although there is no clear role for defibrotide as prophylaxis, even for high-risk patients [22]. 

### Combination therapies with INO for relapsed/refractory ALL

**Fig. 2:**
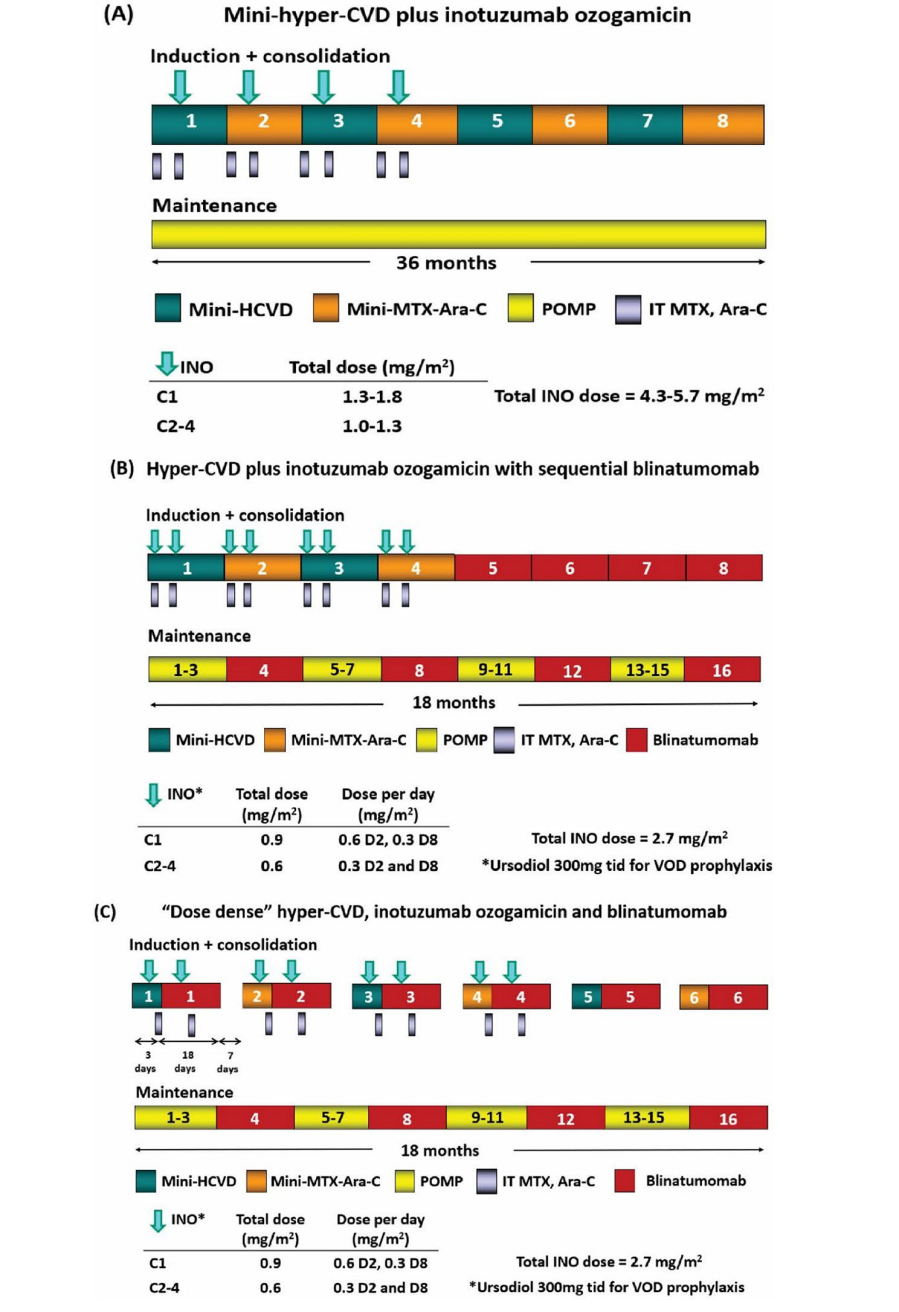
Evolution of the hyper-CVD and inotuzumab ozogamicin ± blinatumomab regimen at MD Anderson Cancer Center. **A**.) Hyper-CVD plus inotuzumab ozogamicin, **B**.) Hyper-CVD plus inotuzumab ozogamicin with sequential blinatumomab, **C**.) “Dose dense” hyper-CVD, inotuzumab ozogamicin and blinatumomab

While single-agent INO therapy represents a therapeutic advance for patients with relapsed/refractory ALL, it is not curative for most patients when given as monotherapy, with < 20% of patients achieving long-term survival [16]. Research efforts have therefore been focused on combination therapies of INO with chemotherapy and/or other novel agents such as blinatumomab, with the goal of deepening response and further improving survival outcomes (Table 2). At MD Anderson Cancer Center, a regimen of mini-hyper-CVD (dose-reduced hyperfractionated cyclophosphamide, vincristine and dexamethasone alternating with dose-reduced methotrexate and cytarabine) in combination with INO was studied in relapsed/refractory Ph-negative B-cell ALL. Figure 2 shows the evolution of this regimen over the past decade. INO was originally given on day 3 of cycles 1–4 at a dose of 1.8 mg/m^2^ in cycle 1 and 1.3 mg/m^2^ in cycles 2–4 (cumulative dose of 5.7 mg/m^2^) and then was later reduced to 1.3 mg/m^2^ in cycle 1 and 1 mg/m^2^ in cycles 2–4 (cumulative dose of 4.3 mg/m^2^) in an effort to reduce the risk of SOS/VOD (Fig. 2A) [23]. Among 59 patients treated, the overall response rate was 78%, with 82% of responders achieving MRD negativity by flow cytometry. Response rates were particularly encouraging in first salvage, where the overall response rate was 91%. The SOS/VOD rate was 15% using this single-dose regimen, which was similar to the 17% rate observed in the initial phase II study using a similar dosing strategy [10]. The median OS was 11 months, and the 1-year OS rate was 46%. The survival outcomes were compared to historical data with INO monotherapy using an inverse probability of treatment weighing analysis, which suggested that the combination therapy was superior to expectations with INO monotherapy.

**Table 2:** Selected ongoing clinical trials of inotuzumab ozogamicin for patients with relapsed/refractory B-cell ALL

Trial	Age (years)	ALL subtypes	Primary endpoint	Data presented or published	Trial Identifier
INO + venetoclax + dexamethasone	18+	Ph-negative or Ph-positive	Maximum tolerated dose	Yes [53]	NCT05016947
INO + augmented BFM	16–60	Ph-negative	Maximum tolerated dose	No	NCT03962465
INO + blinatumomab + mini-hyper-CVD	18+	Ph-negative or Ph-positive	Response rate and overall survival	Yes [25]	NCT01371630
INO induction followed by blinatumomab (Alliance 041703)	18+	Ph-negative	Event-free survival	No	NCT03739814

Abbreviations: INO, inotuzumab ozogamicin; Ph, Philadelphia chromosome

This study was then amended to further reduce and fractionate the dose of INO, add blinatumomab, and mandate ursodiol prophylaxis (Fig. 2B) [24]. The purpose of these changes was two-fold: to deepen response with the addition of blinatumomab and to mitigate the risk of SOS/VOD by reducing the dose of INO and by increasing the interval between the last dose of INO and allogeneic HSCT. In this new design, patients received 4 cycles of mini-hyper-CVD plus INO, followed by 4 cycles of blinatumomab, and then a maintenance phase of blocks of POMP (6-mercaptopurine, vincristine, methotrexate, and prednisone) alternating with blinatumomab. INO was reduced to 0.6 mg/m^2^ on day 2 and 0.3 mg/m^2^ on day 8 in cycle 1 and 0.3 mg/m^2^ on days 2 and 8 in cycles 2–4 (cumulative dose of 2.7 mg/m^2^). In the most recent published analysis of the mini-hyper-CVD, INO ± blinatumomab regimen (with blinatumomab given to patients #68+), 110 patients have been treated [25]. The overall response rate was 83%, and 82% of responders achieved MRD negativity by flow cytometry. The median OS was 17 months, and the 3-year OS rate was 40%. Outcomes were best for those treated in first salvage, where the median OS was 31 months, and the 3-year OS rate was 49%. In a landmark analysis, there was no benefit for receipt of subsequent allogeneic HSCT (3-year OS 54% for both groups). The SOS/VOD rate was also observed to be lower after the amendment to reduce and fractionate INO and add blinatumomab (2% vs. 13% with the previous design; *P* = 0.05). These data highlight that SOS/VOD can be substantially mitigated with use of lower doses of INO without compromising efficacy.

The mini-hyper-CVD, INO and blinatumomab regimen has now been amended to administer to deliver all agents beginning in cycle 1 (Fig. 2C). In the latest study design, 6 cycles of “dose-dense” mini-hyper-CVD, INO and blinatumomab are given, followed by POMP/blinatumomab maintenance in non-transplanted patients. In each cycle, blinatumomab is started on day 4 (i.e. once the mini-hyper-CVD chemotherapy has been delivered) and continues through day 21 of each cycle, followed by a 7-day break before beginning the next cycle. To date, 15 patients with relapsed/refractory ALL have been treated with this regimen. All patients responded, with 92% achieving flow MRD negativity (77% after 1 cycle) [26]. High rates of early response have also been observed in a retrospective analysis of this regimen in both newly diagnosed and relapsed/refractory patients [27]. Among patients with newly diagnosed or MRD-positive ALL, 10/11 (91%) achieved MRD negativity at a level of 10^− 6^ by next-generation sequencing, an endpoint shown to be associated with superior outcomes in ALL [28, 29]. The deep and rapid MRD negative responses with the dose-dose mini-hyper-CVD, INO and blinatumomab regimen are encouraging, and this regimen is also now being evaluated in older adults with newly diagnosed B-cell ALL.

### Combination therapies with INO for newly diagnosed ALL

#### Older adults

Several studies are also evaluating INO in patients with newly diagnosed ALL. Most of these efforts have focused on its use in older adults, a group with poor tolerance to conventional chemotherapy and with historical long-term OS rates of only 20% [31, 32]. Ongoing trials exploring INO in the frontline setting are shown in Table 3, and a summary of available trial data of INO-based regimens in older adults with ALL is shown in Table 4. At MD Anderson Cancer Center, the same mini-hyper-CVD plus INO regimen previously described was also studied in patients ≥ 60 years of age with newly diagnosed Ph-negative B-cell ALL [32]. Initially, 52 patients with a median age of 68 years were treated. The overall response rate was 98%, with 96% of patients achieving MRD negativity by flow cytometry. These high rates of response translated to encouraging long-term survival with 3-year progression-free survival (PFS) and OS rates of 49% and 56%, respectively. As with the relapsed/refractory study, this regimen was later amended to use lower, fractionated doses of INO (cumulative dose 2.7 mg/m^2^), add blinatumomab and mandate ursodiol prophylaxis. A total of 80 older patients have been treated with the mini-hyper-CVD, INO ± blinatumomab regimen (patients #50 + treated with the updated regimen) [33]. Twelve patients (15%) have relapsed, and the 5-year PFS and OS rates are 44% and 46%, respectively. These outcomes compare favorably to the historical 5-year OS rate of approximately 20% when chemotherapy alone is used. The superiority of the mini-hyper-CVD, INO and blinatumomab regimen as compared with dose-reduced hyper-CVAD in a similar older population was confirmed in a propensity score analysis [34]. 

**Table 3:** Selected ongoing clinical trials of inotuzumab ozogamicin for patients with newly diagnosed B-cell ALL

Trial	Age (years)	ALL subtypes	Primary endpoint	Data presented or published	Trial identifier
INO induction followed by low-intensity chemotherapy (INITIAL-1)	56–74	Ph-negative	Event-free survival	Yes [36]	NCT03460522
INO + dasatinib + dexamethasone	18+	Ph-positive	Complete remission with MMR	No	NCT04747912
INO + blinatumomab + hyper-CVAD	14+	Ph-negative	Relapse-free survival	Yes [40]	NCT02877303
INO + blinatumomab + mini-hyper-CVD	60+	Ph-negative or Ph-positive	Progression-free survival	Yes [33]	NCT01371630
INO + mini-hyper-CVD versus dose-adjusted hyper-CVAD (Alliance A042001)	50+	Ph-negative	Event-free survival	No	NCT05303792
INO induction followed by blinatumomab (Alliance 041703)	60+	Ph-negative	Event-free survival	Yes [35]	NCT03739814
INO + low-intensity chemotherapy (EWALL-INO)	55+	Ph-negative	Overall survival	Yes [37]	NCT03249870

Abbreviations: INO, inotuzumab ozogamicin; Ph, Philadelphia chromosome; MMR, major molecular response

**Table 4:** Data from prospective studies of inotuzumab ozogamicin in older adults with newly diagnosed ALL.

Reference (regimen)	*N*	Age in years, median [range]	CR/CRi rate (%)	HSCT rate in first remission (%)	SOS/VOD rate (%)	OS rate (%)
Chevallier P et *al.* 2022 [38] (EWALL-INO: INO + chemotherapy)	131	68 [55–84]	90	8	2	54 (2-year)
Jabbour E et *al.* 2023 [34](mini-hyper-CVD + INO ± blinatumomab)	80	68 [60–87]	99	5	8	46 (5-year)
Stelljes M et *al.* 2024 [37] (INITIAL-1: INO + chemotherapy)	43	64 [56–80]	100	12	2	73 (3-year)
Wieduwilt M et *al.* 2023 [36] (Alliance A041703: INO + blinatumomab)	33	71 [60–84]	96	Not reported	3	84 (1-year)

CR/CRi: complete remission or complete remission with incomplete hematologic recovery; HSCT, hematopoietic stem cell transplant; SOS/VOD, sinusoidal obstruction syndrome / veno-occlusive disease; OS: overall survival; INO, inotuzumab ozogamicin

Despite the improvement over historical expectations, toxicity is still a significant concern with this regimen. Overall, 35 patients (44%) died in remission (including 9 from myelodysplastic syndrome or acute myeloid leukemia, 8 from infection and 5 from SOS/VOD). The risk of death in remission was higher in patients ≥ 70 years of age (accounting for 85% of deaths in remission), resulting in age-dependent survival outcomes (median OS 75 months, 47 months, and 35 months for patients 60–64, 65–69 and ≥ 70 years of age, respectively). Due to the specific risks related to the chemotherapy backbone (e.g. secondary myeloid malignancy and infection), patients ≥ 70 years of age will now receive INO and blinatumomab only, without the mini-hyper-CVD backbone. A similar approach has been evaluated in the Alliance A041703 study [35]. In this trial, patients ≥ 60 years of age with newly diagnosed Ph-negative B-cell ALL received induction with fractionated INO at 1.8 mg/m^2^ in cycle 1 and 1.5 mg/m^2^ in cycle 2, followed by consolidation with blinatumomab for 4–5 cycles. Among 33 patients treated, the overall response rate was 96% (85% after INO induction), and the 1-year OS rate was 84%. Longer term follow-up will be needed to confirm the durability of these responses.

Several other INO-based frontline regimens are being evaluated in older adults with newly diagnosed ALL. In the INITIAL-1 study, patients > 55 years of age with newly diagnosed Ph-negative B-cell ALL received induction with 3 cycles of dexamethasone plus INO (1.8 mg/m^2^ in cycle 1 and 1.5 mg/m^2^ in cycles 2–3), followed by 6 cycles of age-adjusted chemotherapy as consolidation/maintenance.^37^ Forty-three patients were treated with a median age of 64 years (range, 56–80 years). All patients achieved CR/CRi, with 71% achieving MRD negativity at a sensitivity of 10^− 4^ after the 3 cycles of INO induction. The 3-year event-free survival (EFS) and OS rates were 55% and 73%, respectively, and there was only 1 case of non-fatal SOS/VOD. The EWALL-INO study also enrolled a similar population of patients and treated them with 2 cycles of induction consisting of INO, vincristine and dexamethasone (induction 1) and INO, cyclophosphamide and dexamethasone (induction 2), followed by 6 cycles of age-adjusted consolidation and then POMP maintenance [37]. Overall, 131 patients were treated, and the CR/CRi rate after 2 cycles of induction was 90%. The estimated 2-year OS rate was 54%. Taken together, these studies show that frontline INO-based therapy is safe and effective in older adults with B-cell ALL. Building on the promising experience with the mini-hyper-CVD and INO regimen from MD Anderson, the Alliance A042001 is a randomized phase II study evaluating mini-hyper-CVD plus INO versus dose-adjusted hyper-CVAD in older adults (≥ 50 years of age) with newly diagnosed B-cell ALL [38]. No data are yet available, and this study is ongoing.

#### Younger adults

Combination approaches using INO are also being explored in younger adults with newly diagnosed ALL. At MD Anderson, we developed a protocol of hyper-CVAD plus blinatumomab, which has now been amended to add INO. The hyper-CVAD plus blinatumomab regimen consists of 4 cycles of hyper-CVAD, followed by 4 cycles of blinatumomab, and then POMP and blinatumomab maintenance. In the first 38 patients treated, all patients responded, with 97% becoming MRD negative by flow cytometry. This translated to encouraging 3-year relapse-free survival (RFS) and OS rates of 73% and 81%, respectively [39]. An additional 37 patients have now been treated with the addition of INO (0.3 mg/m^2^ on day 1 and 8 of cycles 2, 4, 6 and 8; cumulative dose of 2.4 mg/m^2^) [41]. With a median follow-up of 22 months, only 3 relapses have been observed. The estimated 2-year RFS and OS rates of 88% and 100%, respectively. The initial data with the addition of INO are encouraging and suggest a potential benefit with the routine use of INO in younger patients with newly diagnosed Ph-negative B-cell ALL.

Of note, the Alliance A041501 was a randomized study that also evaluated the addition of INO to standard chemotherapy (CALGB 10,403 backbone) in newly diagnosed B-cell ALL. This study was suspended due to toxicity concerns with the combination regimen, possibly related to the use of multiple hepatoxic agents in this regimen (e.g. INO and asparaginase). The lack of success of this study highlights the need for rationale combinations with INO and to avoid overlapping toxicities.

### Other investigational applications of INO in ALL

#### INO for MRD-positive disease

In the INO-VATE study, INO was associated with a flow MRD negativity rate of 63% among responders [41] and provided support for the evaluation of INO for MRD-positive B-cell ALL. In a phase II study, 26 patients with MRD-positive ALL were enrolled and treated with INO at a dose of 0.6 mg/m^2^ and 0.3 mg/m^2^ on days 1 and 8, respectively, of cycle 1 and 0.3 mg/m^2^ on day 1 and 8 of cycles 2-6 [42]. Sixteen patients (62%) had Ph-positive ALL and also received a BCR::ABL1 TKI (predominantly ponatinib). The MRD negativity response at a sensitivity of 10^− 4^ was 69%, which translated to a 2-year OS rate of 60%. In another study from GIMEMA, INO was evaluated in 20 patients with MRD-positive B-cell ALL. Eleven of 20 patients (55%) achieved MRD response < 10^− 4^ [44]. These encouraging data support the further of evaluation of INO as an MRD-directed therapy in ALL and also provide support for its continued evaluation in the frontline setting to induce deep, MRD-negative remissions.

#### INO for Ph + ALL

INO is active in relapsed/refractory Ph-positive ALL and achieves a CR/CRi rate of 73% and median OS of 8.7 months, which are similar to the findings from the broader population of the INO-VATE study [14]. In a phase I/II study, INO was combined with bosutinib in patients with relapsed/refractory Ph-positive ALL who did not harbor a T315I mutation [44]. Among 18 patients (16 with Ph-positive ALL and 2 with CML in lymphoid blast phase), the CR/CRi rate was 83%, with 56% achieving a complete molecular response. The median OS was 13.5 months, which appears superior to expectations with INO as monotherapy.

#### INO as post-transplant maintenance

INO has been evaluated as post-transplant maintenance in a phase I study of patients with CD22-positive ALL and high-risk for relapse [45]. INO doses of 0.3 mg/m^2^ to 0.6 mg/m^2^ were administered once per cycle for up to 12 cycles. The MTD was 0.6 mg/m^2^. Among 18 treated patients, no cases of SOS/VOD were observed. With a median follow-up of 18.1 months, only 2 relapses were observed, and the 1-year PFS and OS rates were 89% and 94%, respectively. This study suggests that low-dose INO can be safely administered in the peri-transplant setting and may also be helpful in preventing relapse in high-risk patients.

### Sequencing of INO with CAR T-cell therapy

In clinical practice, INO is commonly given prior to CAR T-cell therapy, either as a salvage regimen and as bridging therapy. However, the data are mixed regarding whether prior INO exposure impacts the effectiveness of CAR T-cells [46–48]. Some studies in children have suggested that prior INO—including INO as bridging therapy—did not impact response rates or long-term outcomes following tisagenlecleucel, as compared with historical expectations [46, 47]. However, in the ZUMA-3 study of brexucabtagene autoleucel in adult patients, those with prior INO exposure had numerically lower CR/CRi rates (59% with prior INO exposure versus 77% without prior INO exposure) and inferior OS (median OS 8.8 months and 47.0 months, respectively) [48]. Future studies evaluating the optimal sequencing of INO with other available therapies—including blinatumomab and CD19 CAR T-cells—and the use of INO as bridging therapy prior to CAR T-cell therapy are needed.

## Conclusions

Along with the blinatumomab and CAR T-cells, the clinical development of INO has been a major contributor to improving outcomes of adult ALL over the past decade [49]. While INO has been shown to be more effective than conventional cytotoxic chemotherapy in relapsed/refractory B-cell ALL, its greatest potential is as combination therapy in both the frontline and salvage settings. When used along with low-dose chemotherapy and blinatumomab in relapsed/refractory ALL, a 3-year OS rate > 50% has been observed, even in non-transplanted patients. Similarly, very encouraging outcomes have been observed with INO in newly diagnosed B-cell ALL, whether combined with chemotherapy, blinatumomab or both. Over the course of these studies, the INO dose has been modified, with some studies suggesting that lower, fractionated doses of INO can be highly effective and may also reduce the risk of SOS/VOD, which is one of the feared potential toxicities of INO. Studies continue to expand the potential applications of INO, including its use for MRD-positive disease, combination with BCR::ABL1 tyrosine kinase inhibitors, and its use in low doses as post-transplant maintenance. Many of these ongoing research efforts seek to explore alternative dosing strategies of INO. New translational research is also seeking to understand the mechanisms of resistance to INO, which may help to inform future rational drug combinations [50–53]. The FDA approval of INO in 2017 marked a major milestone that paved the way for these important studies, but it is imperative to note that this was just one step in the clinical development of INO. The research that has followed in the years since the INO-VATE study highlight a truism in oncology: that regulatory approval of a drug is often only the beginning of its true clinical development and innovation.

## Data Availability

No datasets were generated or analysed during the current study.

## Abbreviations

ALL: Acute lymphoblastic leukemia
CI: Confidence interval
CML: Chronic myeloid leukemia
CR: Complete remission
CRi: Complete remission with incomplete hematologic recovery
EFS: Event-free survival
FDA: Food and Drug Administration
HSCT: Hematopoietic stem cell transplantation
INO: Inotuzumab ozogamicin
Mini-hyper-CVD: Dose-attenuated hyperfractionated cyclophosphamide, vincristine and dexamethasone alternating with methotrexate and cytarabine
MRD: Measurable residual disease
MTD: Maximum tolerated dose
NRM: Non-relapse mortality
OS: Overall survival
PFS: Progression-free survival
Ph: Philadelphia chromosome
POMP: 6-mercaptopurine, vincristine, methotrexate, and prednisone
RFS: Relapse-free survival
SOS: Sinusoidal obstruction syndrome
TKI: Tyrosine kinase inhibitor
VOD: Veno-occlusive disease
